# Moral Distress Experienced by US Nurses on the Frontlines During the COVID-19 Pandemic: Implications for Nursing Policy and Practice

**DOI:** 10.1177/23779608221091059

**Published:** 2022-04-07

**Authors:** Shannon D. Simonovich, Kashica J. Webber-Ritchey, Roxanne S. Spurlark, Kristine Florczak, Lucy Mueller Wiesemann, Tiffany N. Ponder, Madeline Reid, Denita Shino, Bonnie R. Stevens, Elizabeth Aquino, Donna Badowski, Christina Lattner, Cheryl Soco, Susan Krawczyk, Kim Amer

**Affiliations:** 1 School of Nursing, College of Science & Health, 2453 DePaul University, Chicago, IL, USA; 2 College of Nursing, 14689 Purdue University Northwest, Hammond, IN, USA; 3 School of Nurse Anesthesia, 3271 NorthShore University HealthSystem, Evanston, IL, USA

**Keywords:** mental health, workforce, qualitative research, research, COVID-19, public health

## Abstract

**Introduction:**

The ongoing COVID-19 pandemic represents the largest contemporary challenge to the nursing workforce in the 21^st^ century given the high stress and prolonged strain it has created for both human and healthcare supply resources. Nurses on the frontlines providing patient care during COVID-19 have faced unrivaled psychological and physical demands. However, no known large-scale qualitative study has described the emotions experienced by nurses providing patient care during the first wave of the COVID-19 pandemic in the US. Objective: Therefore, the purpose of this study was to qualitatively describe the emotions experienced by US nurses during the initial COVID-19 pandemic response.

**Methods:**

One hundred individual interviews were conducted with nurses across the United States from May to September of 2020 asking participants to describe how they felt taking care of COVID-19 patients. All interviews followed a semi-structured interview guide, were audio recorded, transcribed, verified, and coded by the research team.

**Results:**

Participants narratives of the emotions they experienced providing patient care during COVID-19 unequivocally described (1) moral distress, and moral distress related (1.1) fear, (1.2) frustration, (1.3) powerlessness, and (1.4) guilt. In sum, the major emotional response of nurses across the US providing patient care during the pandemic was that of moral distress.

**Conclusion:**

Investments in healthcare infrastructures that address moral distress in nurses may improve retention and reduce burnout in the US nursing workforce.

## Introduction

In early 2020, the world became acutely aware of the spread of the novel Severe Acute Respiratory Syndrome Covid-2 coronavirus (SARS COVID-19). The World Health Organization (WHO) declared a public health emergency of international concern on January 30th, 2020 (WHO Director-General Dr Ghebreyesus, 2020). There were over 1.25 million global deaths, 53% of which are from Regions of the Americas (WHO, 2020). Nurses across the globe were called to action and responded courageously.

## Review of Literature

Nursing practice was challenged by the stress the pandemic placed on healthcare systems and the communities they serve. In the United States (US), there were over 156,000 documented cases of healthcare professionals (HCP) who had contracted COVID-19 between February and September 2020 (National Nurses United, 2020). Among the cases reported from February 2020 to February 2021, over 3,300 nurses, doctors, social workers, and physical therapists have died from COVID-19 (Jacobs, 2021). Nurses who practiced during the pandemic risked their physical health. Nurses also experienced psychological and emotional stressors in the workplace that culminated in moral distress. One of the first studies on nurses by An et al. (2020) reported that frontline nurses in China experienced psychological and mental exhaustion in their role in responding and caring for patients. Among those frontline nurses, 43.6% of the sample population were showing symptoms of depression that impacted their quality of life (An et al., 2020).

Nursing is the heart and soul of healthcare. Even under challenging circumstances, nursing has been acknowledged as the most trusted profession for the past 18 years (Reinhart, 2020). Nursing can be a remarkably stressful profession. According to the Occupational Information Network of the US Department of Labor, the antecedents of nursing stress are the frequency, responsibility and consequences of critical decision making; the physical proximity to adverse, hazardous or unpleasant conditions; and working with potentially infectious specimens and patients (Summary Report, 2020). These stressors were amplified and accentuated during the COVID-19 global pandemic. In the beginning of the pandemic, hospitals were unable to ensure the safety of nurses caring for patients with infectious diseases due to inadequate scientific understanding of the COVID-19 virus and an alarming national shortage of appropriate personal protective equipment [PPE] (Schlanger, 2020).

Prior epidemics such as the SARS-CoV outbreak in 2003 have exemplified the mental strain on nurses that are now being seen with COVID-19 (Maunder et al., 2003). A study that specifically investigated psychological distress in nurses during SARS-CoV reported that nurses who were directly caring for patients with the virus had higher incidences of stress reaction syndrome. Stress reaction syndrome included symptoms of anxiety, depression, hostility and somatization (Chen et al., 2005). Nurses expressed feelings of fear, anxiety, frustration and anger which were prompted by concerns for their families’ welfare, changes in protocols, knowledge of the lethality of the infection, and isolation due to quarantine restrictions (Maunder et al., 2003). A recent qualitive study that specifically investigated causes of moral distress among nurses providing care during the COVID-19 pandemic and identified moral distress causes at individual, relational, organizational, and systematic levels of clinical practice (Silverman et al., 2021). Sezgin et al. (2021) conducted a descriptive qualitive study among nurses working in intensive care units during the COVID-19 pandemic to identify their experiences during this time. Nurses reported increased risk of infection, psychological burden, and lack of professional satisfaction (Sezgin et al., 2021). The burden nurses shouldered during the COVID-19 global pandemic calls for research to describe and examine the emotional wellbeing of nurses during this unprecedented time in contemporary history.

Nurses on the frontlines providing patient care during COVID-19 have faced unrivaled psychological and physical demands. However, no known large-scale qualitative study with a diverse sample of US nurses has detailed the emotions experienced by nurses providing patient care during the first wave of the COVID-19 pandemic. Therefore, the purpose of this study was to describe the emotions felt by US nurses providing patient care during the initial COVID-19 pandemic response in the US.

## Methods

### Design

This qualitative descriptive study examined nurses’ emotions providing patient care during the first wave of the COVID-19 pandemic. We conducted semi-structured one-on-one interviews with nurses across the United States from May 2020 to September 2020, asking each nurse interviewed “How do you feel taking care of COVID-19 patients?” to elicit narrative descriptions of nurses’ emotions.

To obtain broad understanding of the experiences of nurses providing care during COVID-19, an extensive research study team was assembled to aid in both participant recruitment and data collection, with extensive effort exerted to obtain participants from traditionally underrepresented groups in nursing research. More information on the recruitment strategies employed to yield the diverse sample represented in this large-scale qualitative study is available (Webber-Ritchey et al., 2021a; Webber-Ritchey et al., 2021b). The principal investigator was responsible for building the research study team of 24 which at completion included 14 doctorally-prepared nurse scientists and advanced practice nurses from specialties including emergency department, acute care and outpatient settings who meaningfully contributed to participant recruitment, analysis and dissemination efforts. Our study team also included 10 graduate student research assistants who aided in participant recruitment, data verification and analysis efforts. The research team met regularly from May 2020 to date forward to discuss study progress and initial interview findings. As group consensus was determined within the emergency department and acute care nursing specialty areas, efforts were concentrated to recruit nurses from other specialty areas to determine whether there may be new emerging themes that warranted continued exploration. Using Glaser and Straus principles of qualitative study design, this study reached data saturation within multiple subgroups of nurses (Glaser & Strauss, 1967). With funding to complete 100 interviews, the study team made considerable efforts to represent the breadth of nursing experiences during the first wave of the COVID-19 pandemic in the US.

The interview guide was primarily developed by the principal investigator, a PhD-prepared nurse scientist trained in qualitative methodology, based on empirical literature examining nurses’ experiences during infectious disease outbreaks including SARS and Ebola, and in collaboration co-investigator, Dr. Cheryl Soco, an expert Emergency Department Nurse Practitioner with 20 years’ experience working at a major metropolitan level one trauma center during the first wave of the COVID-19 pandemic. The goal of the interview guide was to examine nurses’ experiences caring for patients during the first wave of the COVID-19 pandemic including their preparation, formal and informal forms of support, and coping mechanisms, as well as the impact of the pandemic on the nursing profession, nursing education and policy implications and can be found in its complete state in our previous publication focused on nursing communication during the first wave of COVID-19 (blinded citation). The analysis for this publication details the participants’ narrative responses to the interview guide question, “*how do you feel taking care of COVID-19 patients?”* to describe the emotions experienced by nurses providing care to COVID-19 patients during the initial US response.

Prospective participants were asked to complete a screening tool which assessed their demographic characteristics and area of nursing practice before the interview was scheduled. This enabled our team to intentionally enroll a diverse sample of study participants. All interviews took place via telephone. Nine study team members, including the principal investigator and eight co-investigators, recorded the 100 interviews. Each co-investigator received identical training one-on-one with the principal investigator. Each interview lasted from 20 to 35 min depending upon the length of responses by the study participant. At the beginning of each interview, the study team explained the interview process, reviewed the information sheet, and acquired verbal consent to begin the interview. Given the sensitive nature of these interviews, a waiver of documentation of consent was granted by the IRB. The interviewers were trained to observe for signs of distress and offer to stop the interview if the participants seemed stressed or distressed. The participants were also told that they could stop the interview at any time. Each semi-structured interview used the interview guide for consistency across the study sample. The same open-ended questions were asked in the same order during each of the 100 interviews (citation of study team publication with interview guide here). The principal investigator debriefed with the research team members in weekly meetings during the data collection phase of this study. Audio recordings of each interview were uploaded to a secure cloud-based web application for storage. The Happyscribe platform was used for automatic transcription of the audio recordings. After transcription, each audio recording was verified by hand by trained graduate student research assistants for accuracy.

### Research Question

The research question for this specific examination of the study data was, “What were the emotions experienced by nurses providing patient care during the initial COVID-19 pandemic response in the US?”

### Sample

The sample for this qualitative descriptive study was comprised of self-reported practicing nurses providing patient care during the first wave of the COVID-19 pandemic in the US. The geographic bounds of the study were that participation was limited to nurses actively providing patient care in the United States. The study sample purposefully includes nurses from diverse backgrounds with regards to race, ethnicity, area of nursing specialty, level of nursing education, length of nursing experience and employment settings. We confirmed that all study participants were in nursing roles as of March 2020 when the US pandemic response began and included each participant's nursing employment information and sociodemographic characteristics both during the initial screening process and again at the beginning of their individual interview.

### Inclusion/Exclusion Criteria

Study participants were recruited predominantly through distribution of a digital recruitment flyer through social media platforms including utilization of nursing-specific Facebook groups and Instagram handles. Additionally, nurses were recruited through snowball sampling by word of mouth from other study participants as the study protocol actively asked participants to pass the study information along to colleagues who may be interested in participating. In total, nearly 440 nurses in the US contacted the research team's email address to participate in the study protocol (Webber-Ritchey et al., 2021a). Each prospective participant was screened to assess their individual demographic characteristics as well as their nursing specialty area in order to utilize our 100 interview spots for the most racially and ethnically diverse study sample possible. This focus was important to our team not only for proper representation of the diversity of the nursing workforce, but is also especially critical in our study's aim to capturing nurses’ experiences during a pandemic that has disproportionally impacted communities of color. The principal investigator determined the study eligibility criteria for participants based upon the following inclusion criteria: nurses, including all levels of education and areas of practice, who self-identify as having provided nursing care during COVID-19 and are able to complete the study protocol in English. Exclusion criteria included nurses unwilling to be audio-recorded.

### Institutional Review Board Approval

All data collection processes, procedures, and formal documentation received proper approval from the DePaul University Institutional Review Board (IRB).

### Statistical Analysis

Descriptive analysis of study participant characteristics were tabulated and analyzed by the principal investigator and study coordinator. The qualitative interview data were analyzed using thematic network analyzes first by hand, reviewing the first 20 transcripts as a full team for the emotions experienced by nurses during COVID-19 resulted in identification of moral distress as a global theme across the study sample. Once the thematic network was completed and discussed by the full team, formal purposeful deductive coding was completed using Dedoose's web-based software platform. The analysis for this study was conducted by 9 research study team members (SDS, KJWR, RSS, KF, LMW, MR, DS, BRS, KA), and formally coded by 4 graduate research assistants (TNP, MR, DS, BRS). For the purposes of this study, codes related to the emotions nurses experienced during the first wave of COVID-19 while providing patient care were synthesized into distinct themes related to moral distress.

## Results

### Sample Characteristics

From May 2020 to September 2020, a total of 100 US nurses completed the study protocol. The focus on diversity of participants resulted in a study sample composition in which the majority of nurses interviewed, 65%, identified as a member of a racial, ethnic, or gender minority group first described in our Internaional Nursing Review publication (Simonovich et al., 2021). Employment for our sample of nurses included academic medical centers, 36%, multi-center hospital systems, 17%, independent community hospitals, 16%, outpatient and community-based settings, 23%, federal hospital systems, 5%, and 3% who preferred not to report their employment setting. Specialties represented by our study sample were broad including emergency department, 19%, intensive care, 13%, medical surgical, 13%, labor and delivery, 22%, outpatient/community health, 14%, anesthesia, 2%, leadership, 7%, and 10% of participants who reported multiple specialty areas. Study sample characteristics are presented in full in Table 1.

**Table 1.** Study Sample Characteristics (n = 100).

Age	
Mean	37.9
Range	38
Min, Max	24, 62
Gender	
Female	84
Male	14
Trans/Non-Binary	2
Race	
White	57
Black	20
Asian	14
Multiracial	7
American Indian	2
Ethnicity	
Hispanic	20
Non-Hispanic	80
Education	
Diploma	1
Licensed Practical Nurse	2
Associate's Degree	4
Bachelor's Degree	41
Master's Degree	42
DNP	9
PhD	1
Years of Nursing Experience	
Mean	11.04
Range	41
Min, Max	<1, 42
Employment	
Academic Medical Center	36
Multi-Center Hospital System	17
Independent Community Hospital	16
Outpatient/Community-Based	23
Federal Hospital System	5
Preferred Not to Report	3
Specialty	
Emergency Department	19
Intensive Care Unit	13
Medical/Surgical Nursing	13
Labor & Delivery	22
Outpatient/Community	14
Anesthesia	2
Leadership	7
Multiple Specialties	10

DNP = Doctor of Nursing Practice.

### Research Question Results

**Figure 1.**
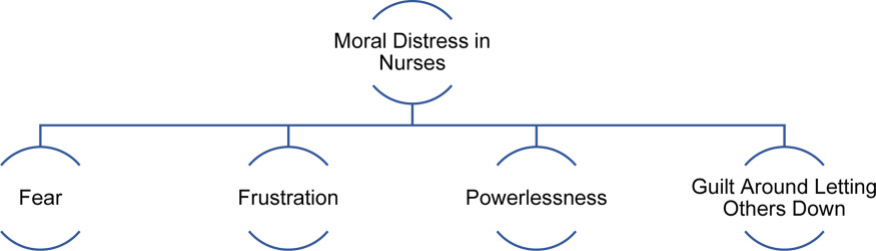
Conceptual framework describing moral distress among US nurses during first wave of COVID-19.

After reviewing all interviews of nurses describing their personal experiences providing care during COVID-19, coding and thematic analysis revealed the major theme of (1) moral distress and four subthemes including (1.1) fear, (1.2) frustration, (1.3) powerlessness, and (1.4) guilt around letting others down. A combination of embedded and long form quotations are presented here to signify the concurrent richness and extensiveness of the participants’ narratives around moral distress. Figure 1 depicts the conceptual framework demonstrating how the unpredictability of changing expectations and nursing practice due to COVID-19 led to moral distress and specific emotional responses in nurses during the first wave of the pandemic in the US. Illustrative quotes for each theme and subtheme are presented in Table 2.

**Table 2.** Key Themes and Illustrative Quotes.

Theme		Illustrative quotes
*1. Moral Distress*		*“I’ll be a nurse thirty-one years at the end of the month and I have never seen the amount of death I have seen in the last three months. And it is beyond emotional and physical. And, you know, it's just you feel so bad for the patient, the family, the staff, because we’re not allowed visitors. So, these patients, a lot of times, are dying without their family present. So, we’re, of course, we’re stepping in and we’ve never let anyone die alone.”* *“And to know that there was just one gown that I wore for the entire shift, we would come out and like spray each other down with bleach and then hang the gown up. And we wore one mask the entire time. It was frightening because we know that's not what's supposed to be done. N95 s were not created for you to wear for eight to 12 h shift… never changing it in between patients [and] just not being able to take it off at all.”* *“I chose to be a nurse practitioner because I love the patient interaction, especially touch…I love the fact that I could touch you and hold your shoulder and say, hey, you’ll be OK, or I would pull this stool in the room, listen to your concerns and address them because that's what I wanted to do. That's what I love to do. Now we’re finding that, not only as nurses are we going through compassion fatigue, but the volume is so tremendous that you’re not able to do that. And then I come back with feelings of guilt. It's not about quality time with the patient anymore, because not only is my cup empty, but there's so many more people to take care of.”* *“[COVID-19 has been] the most emotionally taxing experience of my career as a nurse just because, like me personally, any time I lose a patient, any time something is wrong with a patient, I feel for them… I’m praying for my patients. I cry when they pass. So…to see the overflow of death was traumatizing…That's exhausting to even think about when we get into [nursing it's] because we do care and we want to, you know, make the difference.”* *“It's a new paradigm for nursing to choose between nursing and taking care of yourself.”* *“Yeah. It felt so horrible. It felt like so here I am putting myself at risk, coming to work, taking care of patients and I may contract something… and what's going to happen to me. Who's going to take care of my family? … I think the institutions didn't… really think about the health care workers and their safety. It's scary because it's a lot that's unknown…The anxiety level of knowing that a patient is [COVID] positive is definitely tremendous. There's so much that's unknown. You don't know. You absolutely don't know.”* *“I think we’re going to have massive burnout across this country with…nurses who have been working with COVID patients. And if they don't have a good support network, they’re not going to do well. So, I think that needs to be things in place to have some kind of a tracking system or something to see how people are doing, especially in the midst of a crisis.”* * *
	*1.1. Fear*	*“Well, mentally. I mean, I feel like the mental, the psychological part is just the number one for us in the health care field. And we just don't know. It's the fear of the unknown.”* *“This virus made a lot of older nurses and nurses with preexisting conditions retire. It did instill a lot of fear to the point of the nurses quit[ting] their jobs… It just shook everything.”* *“Nursing [is a] hazardous occupation…It can be quite dangerous, you know…even when we had patients with hepatitis and TB…* *But when you’re dealing with HIV, you can get…prophylaxis. With COVID, it's the waiting game…So it's kind of scary to me, it's kind of dangerous.”* *“I think a lot of people get scared of the idea of taking care of someone who is so infectious, but…nurses are used to dealing with adapting situations… I think the fear was very real and it's gonna stick with people for a long time.”* * *
	*1.2. Frustration*	*“It does make me frustrated that there's no medical people in management. So, it's like people are making decisions, aren't necessarily aware of how it works. Right? Which is always the frustration. But it's really apparent in this situation?…* *Why are we not having more power and more say in things?…* *I’m seeing a lot of gaps that need to be addressed.”* *“I love my job. I love doing patient care…[but] I was not being the provider that I like to be in that first week or two when people were coming in for things that were not urgent, for things that they did not need immediate care for, for things they could have done over the phone or waited, you know, months in some cases, or didn't even need health care for, period. And so, when those patients came in, it was very difficult to ‘mask,’ to use an appropriate term. It was very difficult to mask my frustration with them.”* *“When I first learned of COVID-19, it was kind of downplayed. I guess there were a lot of unsure because it is a fairly new disease. And so…in early March, I remember receiving a letter from our organization, the AANA, which recommended that all health care providers, nurse anesthetists wear N95 masks when participating in intubations and, you know, anything that having kind of respiratory, you know, a risk of respiratory secretions being excreted. And there was a lot of backlash when I went to work saying that we need N95 s. We were told that they had locked the N95 s up and that we did not need N95 masks to provide care and that… we just need a regular surgical mask. And to my dismay… after reading what our organization had told us about the recommendations, a lot of nurses and nurse anesthetists were agreeing with the hospital for fear of backlash, for fear of being fired, for fear of not having work.”* *“People didn't have a lot of knowledge about [COVID-19]. And I felt like… there were a lot of changes and protocols that [were] being made and changes to our hours and operations in general without a lot of consent from employees, which was just really frustrating for a lot of people…So yeah, it was really stressful, lots of adjustment, but lots of changes are happening really fast.”* *“I think [frustration] has been the experience of a lot of nurses. Right? To go through a lot, make a lot of sacrifices, and it's often overlooked.”* * *
	*1.3. Powerlessness*	*“Even when I was there prior to COVID, we were not really involved in…decision making. And it's really unfortunate because the people who govern…nurses are not people who have health backgrounds. So if we make a suggestion, it sort of falls on deaf ears because they’re not health professionals. So how do they know what's important, especially if they’re not listening to us?”* *“I was just very nervous… with a lot of the administrators. I felt like I was powerless because they had these masks and they were locking them up. And, you know, it was almost like, this is all we have.”* *“[I was told by a manager] ‘You know, if you’re going to complain about it, you can find another job’… I really felt very demoralized.”* *“I mean, you didn't have a choice, when this was all happening… you lose your job if you don't come to work. So just knowing that if something like this happens again, you don't have a choice. You have to come to work… unless you want to lose your job, obviously.”* *“So unfortunately, it's the nurse mentality. I feel we kind of just suck it up and we move on. But I know that it's been hard.”* * *
	*1.4. Guilt Around Letting Others Down*	*“I feel bad if I leave because I love the people I work with and so I have a little bit of guilt about abandoning them. But mentally and physically, I don't know how I can keep this up.”* *“There were situations where they were not proning [patients]. So, you would see a patient who would be on high flow nasal cannula and or optiflow and then find them not proning… then you would see them decompensate. You’d be thinking, ‘if they were proned would they get better. Are we like making them worse by not giving them this treatment that we know that could help them?’ So, we would…have these discussions with doctors about that [during] panels [and] debriefs afterwards. We talked about how we did feel like…things were unethical. We did talk to the doctors about it, you know, trying to advocate for patients. But it kind of didn't necessarily feel like we went anywhere with it. We felt like people…wouldn't help…the same thing would happen. Like the patients get intubated and they would eventually pass. And so, they said we could have put an ethics consult in for that. But at that time, it's like so frustrating, you know, that I don't know if we could … have helped.”* *“It's an interesting time… to be a health care provider because usually you don't think there's just this level of fear. I think that didn't exist before… between patient and provider like especially with our pregnant patients, I think they were like, ‘oh my God, I’m going to [go into the] clinic. And if I don't have COVID, I’m going to get it from [the healthcare team].’ And we…felt the same way, like our patients were going to give us COVID. All of a sudden, this person who's coming in for her care could potentially give you an illness that might do nothing to you or it might kill you…it injected this uncertainty into patient provider relationship.”* *“The hardest part…for me, was the separation of the families [from] the patient and the suffering that [it] caused…* *My patients…* *many of them were elderly and…* *their spouses would be sobbing on the phone saying, ‘is there any way you can get me into that room?…* *I’ve been at his side for 65 years….* *Now at this important time, I can't be with him.’ And it broke my heart. It was very hard. So that, for me, was the hardest thing.”* * *

AANA = American Association of Nurse Anesthesiology.

### Theme 1. Moral Distress

Notably, all 100 study participants described moral distress in their one-on-one interviews. Study participants resoundingly articulated a chasm between how they would have liked to have performed according to their professional duty and obligations as nurses versus the reality of providing patient care during the first wave of the pandemic. The chaotic and unpredictable patient care environment during the initial COVID-19 response impacted the ability of nurses to optimally perform their professional roles. The nurses interviewed described experiences of moral distress, previously defined in the literature as a nurse being cognizant of action required in a situation but not being able to carry it out, and the emotions related to this predicament (Corley, 2002; Jameton, 1984). Nurses were simultaneously overwhelmed by the the large number of COVID-19 patients, unknown COVID-19 disease process, the high level of acuity among COVID-19 patients, the rate of patient deaths to COVID-19, and inadequate medical supplies, resulting in significant moral distress among nurses across the country. One participant shared,

I’ll be a nurse thirty-one years at the end of the month and I have never seen the amount of death I have seen in the last three months. And it is beyond emotional and physical. And, you know, it's just you feel so bad for the patient, the family, the staff, because we’re not allowed visitors. So, these patients, a lot of times, are dying without their family present. So, we’re, of course, we’re stepping in and we’ve never let anyone die alone.

Another example of moral distress articulated by study participants was the way in which nurses had to practice with limited supplies, including conservation of personal protective equipment. One nurse shared,

And to know that there was just one gown that I wore for the entire shift, we would come out and like spray each other down with bleach and then hang the gown up. And we wore one mask the entire time. It was frightening because we know that's not what's supposed to be done. N95's were not created for you to wear for eight to 12 h shift… never changing it in between patients [and] just not being able to take it off at all.

Beyond the moral distress of not having adequate medical supplies to protect themselves and service their patients adequately, nurses interviewed also articulated moral distress due to the exhaustion. One participant stated,

not only as nurses are we going through compassion fatigue, but the volume is so tremendous that you’re not able to do that. And then I come back with feelings of guilt…it's not about quality time with the patient anymore, because not only is my cup empty, but there's so many more people to take care of.

Witnessing the moral distress in themselves and their colleagues, one participant shared

nurses who have been working with COVID patients, if they don't have a good support network, they’re not going to do well.

These are all examples of nurses expressing both moral distress in response to changing expectations and nursing practice due to COVID-19. Within the main theme of moral distress, four specific subthemes articulating the emotions felt by nurses experiencing moral distress emerged: (1.1) fear, (1.2) frustration, (1.3) powerlessness, and (1.4) guilt around letting others down.

#### Subtheme 1.1 fear

Study participants resoundingly reported “fear of the unknown” in providing patient care during the first wave of the COVID-19 pandemic. Our first subtheme within moral distress, fear, encapsulates feelings of worry among study participants, particularly the “fear of the unknown” in providing nursing care to COVID-19 patients during the initial pandemic response which was perceived to be “scary” and “dangerous” by study participants. One nurse, senior in their career, shared,

This virus made a lot of older nurses and nurses with preexisting conditions retire. It did instill a lot of fear to the point of the nurses quit[ting] their jobs… It just shook everything…

Study participants across the country shared candidly about the fear of taking care of COVID-19 patients when little was known about the level of contagion or disease process.

I think a lot of people get scared of the idea of taking care of someone who is so infectious, but…nurses are used to dealing with adapting situations… I think the fear was very real and it's gonna stick with people for a long time.

#### Subtheme 1.2 frustration

Our second theme, *frustration*, is defined as nurses’ disquiet in response to unmet needs and feeling unacknowledged. Study participants described many forms of frustration while providing patient care during the first wave of the COVID-19 pandemic from frustration with healthcare leadership to frustration during patient interactions. One nurse interviewed described,

It does make me frustrated that there's no medical people in management. So, it's like people are making decisions, aren't necessarily aware of how it works. Right? Which is always the frustration. But it's really apparent in this situation?… Why are we not having more power and more say in things?… I’m seeing a lot of gaps that need to be addressed.

Another study participant echoed similar concerns in sharing,

When I first learned of COVID-19, it was kind of downplayed. I guess there were a lot of unsure because it is a fairly new disease. And so…in early March, I remember receiving a letter from our organization, the AANA, which recommended that all health care providers, nurse anesthetists wear N95 masks when participating in intubations and, you know, anything that having kind of respiratory, you know, a risk of respiratory secretions being excreted. And there was a lot of backlash when I went to work saying that we need N95 s. We were told that they had locked the N95 s up and that we did not need N95 masks to provide care and that… we just need a regular surgical mask. And to my dismay… after reading what our organization had told us about the recommendations, a lot of nurses and nurse anesthetists were agreeing with the hospital for fear of backlash, for fear of being fired, for fear of not having work.

Nurses also reported disappointment about the level of care provided to their patients as illustrated by one study participant who shared,

I love my job. I love doing patient care…[but] I was not being the provider that I like to be in that first week or two when people were coming in for things that were not urgent, for things that they did not need immediate care for, for things they could have done over the phone or waited, you know, months in some cases, or didn't even need health care for, period. And so, when those patients came in, it was very difficult to ‘mask,’ to use an appropriate term. It was very difficult to mask my frustration with them.

Nurses described feeling excluded or ignored including one participant who shared, *“I think [frustration] has been the experience of a lot of nurses. Right? To go through a lot, make a lot of sacrifices, and it's often overlooked.”* The pandemic stretched healthcare workers and administrators to their capacity. It was challenging to provide care and support to colleagues and patients resulting in frustration among nurses across the US.

#### Subtheme 1.3 powerlessness

Our third theme, *powerlessness*, is defined as the nurses’ inability to influence an outcome and/or voice their concerns. One nurse discussed how they were told by a supervisor that, *“if you’re going to complain about it, you can find another job”* remarking *“I really felt very demoralized.”* One nurse discussed her institution's lack of involvement of nurses in helping develop safety plans stating,

Even when I was there prior to COVID, we were not really involved in…decision making. And it's really unfortunate because the people who govern…nurses are not people who have health backgrounds. So if we make a suggestion, it sort of falls on deaf ears because they’re not health professionals. So how do they know what's important, especially if they’re not listening to us?

Another participant shared that, *“we kind of just suck it up and we move on…I know that it's been hard.”* These statements reinforce the theme of powerlessness felt by nurses providing patient care during the first wave of the COVID-19 pandemic.

#### Subtheme 1.4 guilt around letting others down

Study participants expressed much guilt around letting others down while providing care during the first wave of COVID-19 in which their duty and dedication as nurses and their professional responsibility to provide care for patients was challenged. Our fourth theme, guilt around letting others down, is defined as nurses’ regret surrounding care and decision-making as it relates to themselves, their colleagues, and the treatment of their patients and family units. For example, one nurse shared having *“guilt about abandoning them. But mentally and physically, I don't know how I can keep this up.”* An example of nurses feeling guilt due to moral distress is described in one participant's narrative where they shared,

There were situations where they were not proning [patients]. So, you would see a patient who would be on high flow nasal cannula and or optiflow and then find them not proning… then you would see them decompensate. You’d be thinking if they were proned would they get better. Are we like making them worse by not giving them this treatment that we know that could help them? So we would…have these discussions with doctors about that [during] panels [and] debriefs afterwards. We talked about how we did feel like…things were unethical. We did talk to the doctors about it, you know, trying to advocate for patients. But it kind of didn't necessarily feel like we went anywhere with it. We felt like people…wouldn't help…the same thing would happen. Like the patients get intubated and they would eventually pass. And so, they said we could have put an ethics consult in for that. But at that time, it's like so frustrating, you know, that I don't know if we could … have helped.

Another nurse described,

The hardest part…for me, was the separation of the families [from] the patient and the suffering that [it] caused… My patients… many of them were elderly and… their spouses would be sobbing on the phone saying, ‘is there any way you can get me into that room?… I’ve been at his side for 65 years…. Now at this important time, I can't be with him.’ And it broke my heart. It was very hard. So that, for me, was the hardest thing.

Study participants broadly described being placed in difficult patient care experiences that resulted in guilt around letting down patients, their families, in addition to fellow members of the healthcare team.

## Discussion

This qualitative descriptive study articulates how providing patient care during the first wave of the COVID-19 pandemic led moral distress in nurses including feelings of fear, frustration, powerlessness, and guilt around letting others down. These study findings embody Jameton's definition of moral distress described as the “negative state of painful psychological imbalance experienced when nurses make a moral decision but cannot act accordingly, because of real or perceived institutional constraints” (Jameton, 1984; Jameton, 1993). In patient care roles during COVID-19, nurses have faced the dilemma of knowing the morally right course of action to take but is blocked by institutional structure and conflicts with co-workers (Jameton, 1984; Jameton, 1993). Providing care to patients during the first wave of the COVID-19 pandemic undoubtedly led to moral distress among the interviewed nurses across the US. Related literature on emotional distress notes that when such states of “mental anguish” exist in overwhelming and stressful work environments, nurses are at risk for emotional suffering including anxiety and depression (Kandola, 2020). The key distinction between moral and emotional distress is that moral distress occurs when a nurse is unable to implement morally right correct action as perceived by their individual nursing judgment (Epstein & DeLgado, 2010). The findings of this qualitative descriptive study are a call to action to better understand and address moral distress in the US nursing workforce.

Extant literature supports COVID-19 pandemic as a traumatic stressor event that is capable of eliciting post-traumatic-like symptoms and exacerbating mental health illness (Bridgland et al., 2021). Fear of uncertainty and fear of being infected have been the most common psychological issues reported in an extensive analysis of literature on health care workers mental health and coping during past epidemics (Cabarkapa et al., 2020). Professional and ethical values were the driving forces toward working throughout the MERS epidemic. The authors stressed the importance of identifying resilience factors that can help maintain the mental health of health care workers. This finding is consistent with a recent study conducted among nurses providing care for patients with COVID-19 (Silverman et al., 2021). Silverman et al. (2021) revealed the need to gain clarity on resiliency factors. Silverman et al. (2021) identified how a supportive ethical climate that includes non-hierarchical interdisciplinary spaces where all providers meet together is necessary to restore nurses’ moral resiliency, which is having the courage and confidence to confront distressful and uncertain situations by following and trusting one's values and beliefs (Hossain & Clatty, 2021).

Burston and Tuckett systematically reviewed literature identifying individual characteristics, site-specific systems, and broader external influences as factors that contribute to moral distress. Silverman et al. (2021) found the major causes of moral distress among 31 US nurses providing care to patients with COVID-19 are: lack of knowledge and uncertainty regarding how to treat a new illness; being overwhelmed by the depth and breadth of the COVID-19 illness; fear of exposure to the virus leading to suboptimal care; adopting a team model of nursing care that caused intra-professional tensions and miscommunications; policies to reduce viral transmission (visitation policy and PPE policy) that prevented nurses to assume their caring role; and practicing within crisis standards of care and dealing with medical resource scarcity. A recent cross-sectional study conducted among nurses working at a University Medical Center in South West Ethiopia found sex, working hours, professional commitment, autonomy, and working environment to be predictors of moral distress (Beyaffers et al., 2020). A descriptive, correlational study found job satisfaction, practice environment, and age as predictors of moral distress among a sample of US critical care nurses (Hiler et al., 2018). The evidence is weak on interventions that reduce moral distress as a result of the different causes and effects of moral distress (Imbulana et al., 2021; Morley et al., 2021).

In Corley's theory of moral distress, a nurse experiences distress when there is insufficient staff and organizational support is lacking resulting in the inability of the nurse to meet the needs of patients (Corley, 2002). Initial distress results in frustration, anger and anxiety and is then followed by moral distress. The nurse's unresolved moral distress can in turn result in suffering, which leads to resignation, burnout, and potentially, leaving the nursing profession (Corley, 2002). Similarly, in a survey among 389 critical care providers, Fumis et al. (2017) found moral distress significantly associated with severe burnout. With this knowledge, a high priority is for the provision of supports at the organizational, unit, and individual level to ensure nurses are empowered to deal with circumstances that precipitate moral distress (Dacar et al., 2019). Additionally, fair reward mechanisms should be provided to acknowledge the contribution of nurses in management of COVID-19 pandemic coupled with hospital administration performing risk assessments at early stages to identify nurses’ needs (Sezgin et al., 2021). Given the economic cost of nursing turnover and the necessity of having sufficient number of experienced as well as trained nurses on the frontlines, nursing leadership must identify and provide viable interventions to nurses to help cope with moral distress (Dacar et al., 2019; World Health Organization, 2017).

### Strengths and Limitations

This qualitative descriptive study represents the first known examination of nurses’ experiences with moral distress during the COVID-19 pandemic. Our timely study protocol allowed us to capture the first-hand accounts of nurses from a wide variety of practice settings and specialty areas in real time as COVID-19 surged throughout the United States. This study has limitations in that the findings articulated by the 100 nurses interviewed may not be generalizable to the full population of nurses working across the country during the pandemic. While robust recruitment measures were in place to aid in participation among nurses from many backgrounds and practice areas, the study team were not successful recruiting many nurses working in long-term care settings. Furthermore, this study relied fully upon self-report data. Self reports are subjective and can be influenced by the participants’ abilities to recall their lived experiences, which can be particularly challenging during periods of high stress. Nevertheless, due to the large sample size and inclusion of emergency, intensive care, and medical surgical nurses, the study team feels that the findings presented are authentic expression of many nurses’ first wave experiences. Future research on moral distress in nursing should include development and testing of interventions meant to assist nurses in reducing their moral distress and improve their processing COVID-19 patient care experiences. Future research should also include interdisciplinary projects in high acuity in-patient settings that work to prevent moral distress in nurses and other frontline workers. Future policy work should include introduction of programs that encourage nurses in leadership positions to aid in supporting front line nurses and involving nurses in decision making. Retention of the nursing work force can be a priority by enhancing the support for nurses experiencing moral distress in the aftermath of the COVID-19 pandemic.

### Implications for Practice

This qualitative study gives voice to the contemporary challenges facing the nursing workforce in a COVID-19 world. This study reflects nurses’ needs for opportunities for self-advocacy, patient advocacy, decision making, and formal emotional support from their organizations, unit leaders and colleagues. A call to action resonates with the need to decrease occurrences of unresolved moral distress among nurses by providing support networks. Failure to engage for the greater good of nurses’ well-being, may hasten nursing professionals’ losses through resignations and burnout. In a profession where there is a code of ethics that speaks to the expectations of advocating for the rights, health, and safety of patients, the same code must speak to the same duty to oneself and one's colleagues. This study's findings articulate a clear need for promotion of psychological resilience in nurses as well as efforts to reduce the long-term effects of moral distress through universal implementation of screening tools such as Psychological First Aid (PFA) (Owen & Schimmels, 2020).

A potential solution is for healthcare organizations and policy makers to make intentional investments in programs that will aid in alleviating moral distress among the nursing work force. A focus on solutions for individual nurses should be implemented promoting emotional wellbeing, regular scheduled breaks with a nurse relieving the staff nurse, paid time off to restore mental and physical health, peer support groups and self-care. For the leadership team, open communication, moral support and opportunities for nurses in decision making roles may improve nursing retention, reduce burnout, and optimize nursing practice improving clinical outcomes for patients and ensuring the health for our communities.

## Conclusions

This study describes the first known large-scale qualitative depiction of moral distress, and the subthemes of fear, frustration, powerlessness, and guilt around letting others down, as experienced by a diverse group of nurses providing patient care across the United States during the first wave of the COVID-19 pandemic in the 2020. Based upon these findings, healthcare policy and practices should assess and address moral distress in the US nursing workforce to improve retention and reduce burnout. Investments to address and prevent further moral distress at this time will help us to be better prepared for future challenges yet to be faced by the 21^st^ century nursing workforce.
